# Supplementary material for: 3D-Cultured Adipose-Derived Stem Cell Spheres Using Calcium-Alginate Scaffolds for Osteoarthritis Treatment in a Mono-Iodoacetate-Induced Rat Model
Source: Int J Mol Sci. 2023 Apr 11;24(8):7062. doi: 10.3390/ijms24087062 (PMC10138691; doi:10.3390/ijms24087062)
Supplement: Supplementary file 1 [file ijms-24-07062-s001.zip › ijms-2318894-supplementary.pdf]

Supplementary Materials

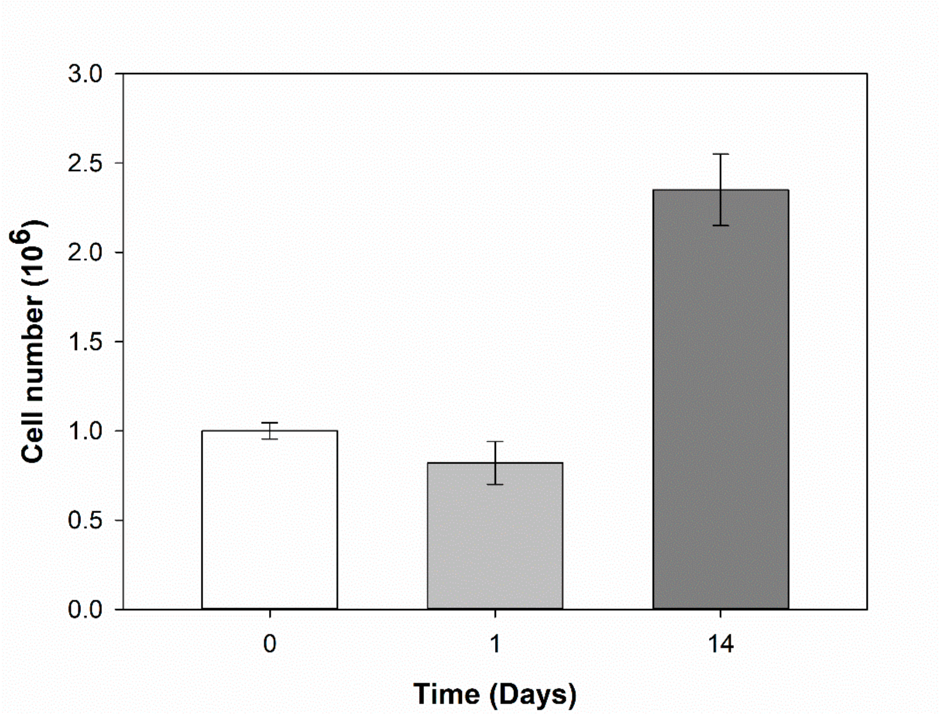

Figure S1. Cell proliferation of hADSCs in Ca-Ag scaffold was analyzed at 0, 1 and 14 days by the CellTiter-Glo® luminescent cell viability assay.

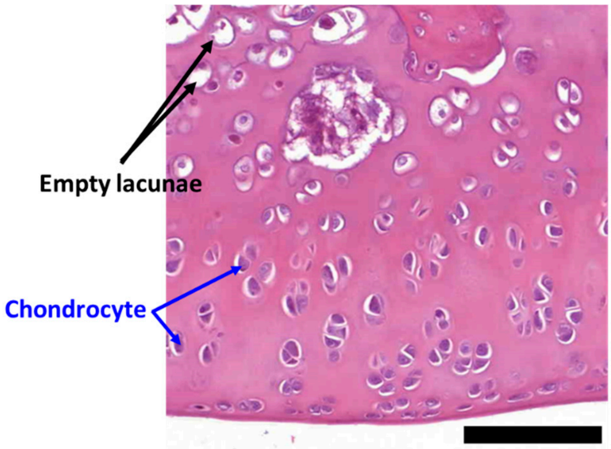

Figure S2. Articular cartilage of the rat knee with H & E staining. The empty lacunae and chondrocyte were indicated by black arrow and blue arrow, respectively (scale bar = 50 μm).
